# Supplementary material for: Are carbon emissions trading and green financial instruments synergistic? -Comprehensive quantitative research based on content analysis
Source: PLoS One. 2024 Mar 7;19(3):e0298601. doi: 10.1371/journal.pone.0298601 (PMC10919601; doi:10.1371/journal.pone.0298601)
Supplement: S1 Data — (ZIP) [file pone.0298601.s001.zip › Data/地级市Z_F工作报告.zip!/╜¡╦╒/╒≥╜¡╩╨/└┤╫╘╣1⁄2╓┌║┼í╛┬φ┐╦╩2╛▌═°í┐_2017.docx]

各位代表：

　　现在，我代表镇江市人民政府向大会作工作报告，请予审议，并请各位政协委员提出意见。

一、本届政府工作回顾

　　本届政府组建以来，全面贯彻党的十八大和十八届三中、四中、五中、六中全会精神，以习近平总书记“镇江很有前途”的殷切寄语为强劲动力，在省委、省政府和市委的坚强领导下，紧紧围绕“强富美高”新镇江建设目标，团结和依靠全市人民，努力践行创新、协调、绿色、开放、共享的发展理念，坚持生态领先、特色发展，推动全市经济社会发展取得了新成就。

　　——五年来，我们坚持稳增长调结构，综合实力迈上新台阶。地区生产总值、固定资产投资、一般公共预算收入分别是2011年的1.66倍、2.34倍、1.61倍。形成高端装备制造、新材料两个千亿级产业，建成北汽华东基地、惠龙易通等一批产业项目，年销售超百亿工业企业达7家。获批全省唯一的国家航空产业示范基地、全省首家国家军民结合产业示范基地。创成国家级高新区、国家现代农业示范区。国家级高新技术企业总数突破600家，高新技术产业产值占比连续四年全省第一，城市创新力列全国第19位。三个辖市均入选“中国中小城市综合实力百强县市”。

　　——五年来，我们不断提升城市品质，城乡面貌发生新变化。市区累计完成城建投资2900亿元，常住人口城镇化率由63%提高到69.2%。连镇铁路、长江-12.5米深水航道开工建设，官塘桥快速路、五凤口高架等一批节点工程竣工通车。成为首批国家海绵城市建设试点。市区完成旧城区城中村改造728万平方米，改造提升老旧小区100万平方米。各辖市区主城建设彰显特色。创成3个美丽宜居镇、30个美丽宜居村庄。

　　——五年来，我们坚持先行先试，低碳镇江成为新品牌。率先在全国探索“四碳创新”(1)，作为中国城市代表，参加中美智慧型低碳城市峰会、联合国应对气候变化巴黎大会。持续开展“三个片区”(2)“一湖九河”(3)环境综合整治。成为全国首批生态文明先行示范区、国家生态市、国家森林城市和全省唯一的生态文明建设综合改革试点市，荣获中国人居环境奖。

　　——五年来，我们不断深化改革开放，发展活力得到新增强。率先实行项目审批“多评合一”(4)和区域评估，成为全国样板。商事制度改革稳步推进。新增市场主体15万户，新增上市挂牌企业103家。新一轮政府机构改革顺利完成。公车改革全面实施。教育综合改革、医药卫生体制改革不断深化。累计实际到位外资92亿美元，国家级综合保税区通过验收，创成2个国家级出口基地。

　　——五年来，我们持续加大民生领域投入，群众生活得到新改善。可用财力的70%、新增财力的80%以上用于民生改善。城乡居民人均可支配收入分别是2011年的1.57倍和1.63倍。累计新增就业41.97万人，转移农村劳动力11.3万人。“百村万户”双达标行动实现既定目标。居民养老保险实现城乡统一；城乡低保标准分别比2011年提高54.3%和166%。新增养老床位1.16万张。全国义务教育发展基本均衡县（市、区）“满堂红”。城乡居民健康素养综合水平连续五年全省第一。文化广场标准化建设入选全国文化创新工程。成功举办中超、CBA(5)、世乒赛选拔赛等大型赛事。居民消费价格指数涨幅控制在3%。

　　——五年来，我们积极创新管理方式，社会建设实现新提升。历经12年持续努力，创成全国文明城市。“大爱镇江”精神广泛弘扬，造血干细胞捐献工作走在全省前列。城乡和谐社区达标率分别达97.5%和98.7%。新增社会组织2031家。食品药品安全“161”社会共治模式(6)全国推广。法治建设满意度和公众安全感全省领先，荣获 “长安杯”(7)。

　　各位代表，刚刚过去的一年是本届政府收官之年。我们认真贯彻中央和省、市委决策，按照“六个年”(8)的总体部署，坚决落实“实现新发展，一切在于干”的要求，积极应对经济下行压力，扎实做好稳增长、调结构、促改革、惠民生、防风险各项工作，较好完成了市七届人大五次会议确定的目标任务，“十三五”发展实现良好开局。

　　经济发展稳中有进。完成地区生产总值3833.8亿元，增长9.3%，增幅列全省第3位；一般公共预算收入293亿元，同口径增长4.7%；固定资产投资2873.4亿元，增长15.1%，增幅列全省第4位；社会消费品零售总额1236.8亿元，增长11.1%，增幅列全省第5位；R&D经费支出占GDP比重达2.59%，列全省第5位；城乡居民人均可支配收入达41794元和20922元，分别增长8.1%和8.9%。

　　产业结构优化提升。新兴产业完成销售4090亿元，增长12.5%，占全市工业销售比重达46.2%。落实《中国制造2025镇江行动纲要》。成功举办“百家央企镇江行”活动。全面实施质量强市战略。新增上市挂牌企业37家，花王股份成功登陆上交所主板。服务业增加值占GDP比重达47.6%，提高0.7个百分点。成功举办香港现代服务业招商会。入围“国家全域旅游示范区”创建单位。城市新地标苏宁广场对外营业。高效设施农业占比达20%。20个产业“三集”示范园区加快建设，首批8家市级特色小镇启动创建。

　　创新创业力度加大。镇江新区综合考评列全省国家级开发区第6，比上年上升12位。镇江高新区省内综合排名上升3位，创新核心区一期启动规划编制。苏南国家自主创新示范区完成“一区十四园”(9)空间布局规划。丹阳市获批省级高新区。成为国家专利质押融资、专利保险“双示范”城市。创成省级以上博士后科研工作站54家，其中国家级21家。新增134家国家级高新技术企业，高新技术产业产值占比达49.1%。实施首期“金山英才”计划(10)，新引进各类高层次产业领军人才130人。开展“创业镇江”行动，镇江创业园被认定为国家级创业孵化示范基地，南京大学国家级双创示范基地落户句容。完成民间投资2035亿元，增长21.5%；新开业各类市场主体4.6万户、注册资本1294.9亿元，分别增长32.9%和27.3%。

　　改革开放深入推进。扎实推进供给侧结构性改革，制定实施“1+5X”政策(11)，淘汰落后产能生产线项目43个，化解水泥过剩产能110万吨，完成“地条钢”生产企业整治；市区商品住房去化周期降至10个月；多措并举为企业减负85.2亿元；发行各类债券473.8亿元，直接融资突破400亿元。深化行政审批制度改革，建成“e办事”网上审批服务平台。深化商事制度改革，企业登记实现“一口受理、一表申报、同步审批”。农村土地承包经营权确权登记颁证工作基本完成。不动产统一登记全面实施。扩大对外开放，完成产业类实际利用外资13.1亿美元；进出口总额103.2亿美元，增长2.5%，增幅列全省第2位。创成国家级服务外包示范城市。

　　生态建设成效显著。以“低碳新经济—技术创新·共享低碳”为主题，成功举办首届镇江国际低碳技术产品交易展示会。国家级生态城镇化示范区正式获批，丹阳创成国家级循环经济示范市，扬中成为全国高比例可再生能源示范市。与中美绿色基金签订3个合作项目。全面推进中央环保督察组交办事项，启动“263”专项行动(12)，实施97个生态环境整治重点项目，全市空气质量优良天数占比达76.4%，PM2.5浓度降至50微克/立方米以内，地表水国考断面优于Ⅲ类水质比例达75%。

　　城乡建设步伐加快。启动“精美镇江”建设三年行动计划。实施6大类、142个城建项目，完成年度投资398亿元。312国道南移工程试通车，九华山路改造建成通车，焦山路全线贯通，丁卯桥路快速化改造按序时推进。海绵城市建设通过中期评估。市区完成棚户区改造93万平方米、危房解危5.3万平方米；建成保障房7154套。完成大港水厂一期主体工程。改造城市干道燃气管道42公里。深入开展城市环境综合整治，创成省级示范路3条、示范社区3个。主城区实现清扫保洁市场化全覆盖。顺利通过国家卫生城市复审。

　　社会民生保障有力。82个民生项目如期完成。开展“百村万户”达新标、精准帮扶特困家庭奔小康、万个党支部结对帮扶万户特困家庭三大行动，74个经济薄弱村达标、4610户农村低收入人口脱贫。企业退休人员养老金实现“十二连增”，城乡低保一体化走在全省前列。机关事业单位养老保险制度改革启动实施。学前教育成为全国36个改革发展实验区之一，镇江市青少年校外综合实践基地建成运行，江苏航院建成招生。完成基层医疗卫生机构标准化建设，建成全国百强社区卫生服务中心2家、国家群众满意卫生院6家、省级示范村卫生室11家。二孩政策全面实施。基层综合文化服务中心建设得到中央改革办肯定，多媒体舞台剧《春江花月夜：赛珍珠》在国内外巡演。成功举办首届江苏航空体育旅游季，里约残奥会镇江籍运动员荣获四金二银的好成绩。城乡社区居家养老服务中心覆盖率分别达100%和96%。民生“三安”(13)持续加强，基本建成危化品防范处置“五大体系”(14)，在全省率先开展食品药品安全“智慧”监管。“七五”普法规划全面启动。实现全国双拥模范城创建“七连冠”。蝉联“全国慈善七星级城市”。民族宗教工作扎实推进，江苏佛学院成功落地。档案、地方志、防震、科普、港澳台交流、外事等工作取得新成绩，老龄、妇女儿童、关心下一代、残疾人、红十字等事业取得新进展，国家安全、国防教育、人民武装、人民防空等工作取得新成效。援藏援疆、南北共建工作走在全省前列，援疆干部王华同志的先进事迹被央视《新闻联播》《焦点访谈》以及《人民日报》等重要媒体报道。

　　自身建设不断加强。扎实开展“两学一做”(15)学习教育，对查摆出的问题逐一整改，研究出台加强政府性债务管理、财政预算追加、经营性用地调控等多项制度。认真落实“三重一大”(16)决策制度，坚持重大事项向市委报告。依法接受市人大及其常委会的法律监督和工作监督，主动接受市政协的民主监督，充分发挥各民主党派、工商联、无党派人士以及人民团体的参政议政作用。承办人大代表建议178件、政协委员提案406件，满意和基本满意率达100%。建立定期学法制度，政府法律顾问工作实现常态化。严格执行中央“八项规定”、省市“十项规定”，政风行风持续好转。

　　各位代表，过去的五年，发展成就令人鼓舞，奋斗历程催人奋进。我们深切体会到:只有坚持以人民为中心，努力让人民拥有更多获得感，才能赢得全市人民的信任和拥护；只有坚持以创新为动力，着力转换发展动能，才能实现新常态下的新发展；只有坚持以绿色为底色，坚定打好生态牌、低碳牌，才能走出镇江特色发展之路；只有坚持以实干为导向，凝聚调动一切力量，才能不断把镇江各项事业推向前进！

　　奋斗饱含艰辛，成绩来之不易！这些成绩的取得，是在省委、省政府和市委的坚强领导下，在市人大、市政协的监督支持下，全市人民同心同德、齐心协力干出来的。在此，我谨代表市人民政府，向全市人民，向各位人大代表、政协委员和离退休老同志，向市各民主党派、工商联、无党派人士、人民团体和社会各界，向驻镇人民解放军、武警官兵和人民警察，向垂直部门和驻镇单位，向所有关心支持镇江发展的海内外朋友，致以崇高的敬意和衷心的感谢！

　　在总结成绩的同时，我们也清醒认识到，发展中还存在一些深层次矛盾和问题，主要是：产业“成点不成链”，高端装备制造、新材料等主导产业链条不长；投资效率不够高，经济发展尚未从投资拉动转向创新驱动；新业态、新技术、新模式比较少，企业内生动力不足，转型升级任务繁重；中心城区对辖市的带动力不强，人口老龄化较为严重；城乡居民持续增收压力较大，部分地区生态环境问题较为突出，保障和改善民生仍需做大量工作；政府的创新能力、执行能力、服务意识、办事效率有待进一步提高。我们一定高度重视这些问题，采取更加有力措施，切实加以解决。

二、今后五年的总体要求和工作思路

　　根据中共江苏省第十三次党代会和镇江市第七次党代会精神，建议新一届政府工作的总体要求是：全面贯彻党的十八大以来中央决策部署以及习近平总书记系列重要讲话精神，紧紧围绕“五位一体”总体布局、“四个全面”战略布局，适应和引领经济发展新常态，自觉践行新发展理念，牢牢把握“稳中求进”工作总基调，扎实推进供给侧结构性改革，坚持“生态领先、特色发展”战略路径，奋力谱写“两聚一高”镇江篇章，向着建设“强富美高”新镇江的宏伟目标开拓前进。

　　建议新一届政府的工作思路是：

　　咬定一个目标：高水平全面建成小康社会。其核心要义是发展质量更高、群众获得感更强。聚力创新，切实提高经济发展的质态；聚焦富民，让发展成果惠及全体百姓，确保小康路上“一个不少、一户不落”。全面完成“十三五”规划，实现地区生产总值、城乡居民人均收入2020年比2010年翻一番，居民收入、企业利润、财政收入“三个口袋”更加充实。

　　把握一个基调：稳中求进。把“稳”的基础打得更牢，稳住经济运行、稳住物价水平、稳住市场预期、稳住社会大局，坚决守住社会民生、生态环境、金融安全等底线；把“进”的步伐迈得更实，在产业发展、科技创新、改革开放、民生实事等方面取得新突破。

　　突出一条主线：供给侧结构性改革。深入推进去产能、去库存、去杠杆、降成本、补短板五大任务，减少无效和低端供给，扩大有效和中高端供给，全面提高发展的质量和效益，推动经济保持中高速、产业迈向中高端。

　　抓住一个机遇：宁镇扬一体化。未来五年，镇江面临着长江经济带开发、扬子江城市群打造、苏南国家自主创新示范区建设等一系列重大战略机遇，但最直接、最现实的机遇就是宁镇扬一体化。我们要在基础设施、产业布局、公共服务、文化旅游、生态保护“五个一体化”上有实质性进展，推动城轨互联、区域共建、资源共享，实现主枢纽对主枢纽、主城区对主城区、主景区对主景区的有效对接，充分承接省会城市的溢出效应，提升镇江中心城区的集聚能力。

　　坚持一个路径：生态领先、特色发展。把生态作为镇江最大的特色、优势和财富，以生态彰显城市魅力，以生态吸引人气和资源，以生态促进经济社会发展。坚持有所为、有所不为，按照苏南现代化建设示范区的定位，保持战略定力，努力建成全国重要的高端装备制造基地、新材料产业基地、区域物流基地、技术研发基地、创意生活休闲中心，成为现代山水花园城市和旅游文化名城。

　　蓝图在前，使命在肩。做好今后五年工作，必须认清形势，把握趋势，发挥优势，进一步焕发“镇江能行”的发展自信。

　　——发挥区位优势，焕发发展自信。镇江江河交汇，因漕运而兴，自古就是商埠重镇。如今，“铁、公、水、空”综合交通优势日益显现。我们必须以更加开放的姿态，吸聚资金、人才、技术、信息等各类资源，形成和扩大镇江发展的动能。

　　——发挥生态优势，焕发发展自信。镇江“有山有水有故事”，资源禀赋得天独厚，素有“城市山林”的美誉。生态是镇江的底色，我们必须以更大力度保护好生态，进一步释放生态红利，让绿水青山成为金山银山。

　　——发挥文化优势，焕发发展自信。镇江3000多年的悠久历史，孕育了灿烂的文化，人文荟萃、人杰地灵。我们必须以敬畏历史、无愧先人的责任和担当，书写新的历史，开辟新的未来。

　　——发挥团结优势，焕发发展自信。镇江的老百姓淳朴勤劳，镇江的企业家充满活力，镇江的干部务实进取。大家都盼望镇江能有更好的发展、更大的进步，这是最宝贵的力量、最强大的潜能。

　　我们坚信，通过五年接续奋斗，宏伟的目标、美好的蓝图，一定会变为现实！

三、2017年的主要任务

　　今年是党的十九大召开之年，是供给侧结构性改革的深化之年，是全面落实市党代会部署的开局之年，也是新一届政府履职的第一年。千里之行，始于足下。我们必须从头抓紧，扎实奋斗。

　　今年经济社会发展的主要预期目标是：地区生产总值增长8%左右；一般公共预算收入同口径增长4%左右；固定资产投资增长12%左右；社会消费品零售总额增长10%左右；R&D经费支出占GDP比重达2.61%；城乡居民人均可支配收入与经济增长同步；全面完成省下达的节能减排约束性指标。

　　工作中，将按照市委七届二次全会部署的“十个更大进展”(17)，着力抓好八个方面：

　　（一）着力振兴实体经济

　　实体经济占全市经济总量的80%以上，是全市经济社会发展的重要根基，必须高度重视、倍加珍惜。

　　一是全力以赴抓项目。以项目为主抓手，坚定不移做强制造业特别是先进制造业。一手抓重大产业项目，实施200个市级重点产业项目，完成投资810亿元。一手抓技术改造项目，重点实施50个制造装备提升项目、107个工业技改项目，推动传统产业向中高端迈进。按照项目清单，排实责任清单，落实“五个一”推进机制(18)，确保项目达序时、见实效。

　　二是全力以赴抓招商。把招商引资摆在更加突出的位置。围绕“产业成链”抓招商。聚焦“海陆空”(19)、新材料、生物医药等产业，以龙头企业为核心，着力引进上下游强链延链补链企业，拉长产业链条，培植产业集群。围绕“军民融合”抓招商。结合争创国家级军民融合创新示范区，本着“互利互惠、合作共赢”的原则，突出通航产业、电子信息、交通装备等重点，积极面向军工集团开展招商，着力引进和培育一批军转民、民参军项目。围绕“国企央企”抓招商。每季度组织《中国制造2025》专题招商，瞄准国企央企，实施精准招引。

　　三是全力以赴促民营。民营经济是我市经济的主体、财税贡献的主力和就业的主渠道。一手抓“顶天立地”，充分发挥大企业的支撑作用，引导具备条件的企业进入资本市场，新增上市挂牌企业25家。弘扬“工匠精神”，支持企业争创国家和省质量奖，创成丹阳五金工具国家级示范区，新增省级以上品牌30个，培育30家行业单打冠军。一手抓“铺天盖地”，顺应大众创业、万众创新的新趋势，降低准入门槛，加强政策扶持，更多释放民间资本活力，新开业各类市场主体4万户、注册资本1000亿元。

　　四是全力以赴优服务。企业家是稀缺资源，生产要素只有在企业家手中，才能变成社会财富。引导全社会尊重企业家、关心企业家、爱护企业家，为企业家创大业、创新业营造更好环境。加强经济运行监测，研究制定重点行业和特困企业精准帮扶政策。强化资金等要素保障，扩大小微企业“周转贷”(20)规模，建立完善“投贷联动”(21)支持政策。树立“亲”“清”政商关系，扎实开展“暖企惠民”大走访，有效破解成本上升盈利难、负担过重生存难、实力不强转型难等实际问题，推动企业加快成长。

　　（二）着力培育创新经济

　　创新是新常态下引领发展的第一动力。坚决摒弃高投入、高能耗、高污染、低效益的粗放式增长方式，集中力量培育创新型增长点，加快产业转型升级。

　　一是布局新产业。结合中国制造2025苏南城市群试点建设，做大做强高端装备制造和新材料两大支柱产业，统筹发展新能源、新一代信息技术、生物技术与新医药等战略性新兴产业，新兴产业销售增长12%。每个辖市区分别确定1-2个主攻方向。丹阳市依托恒神碳纤维、鱼跃医疗等骨干企业，重点发展新材料、生物技术与新医药产业；句容市结合“江苏硅谷”的打造，加快发展大数据、新能源产业；扬中市依托新能源产业园、智能电气产业园，稳步发展新能源和高端化、智能化工程电气产业；丹徒区依托北汽华东基地，重点发展高端装备制造业；京口区以爱励铝业、鼎胜新材为骨干，加速壮大高性能铝材料产业；润州区依托船舶与海工配套产业园、睿泰产业园及惠龙易通，重点发展船舶海工、数字出版与智慧物流产业；镇江新区依托航空产业园、新能源产业园，重点发展航空航天和新能源产业。镇江高新区进一步理顺管理体制，实施产业投资和科技金融项目10个以上，加快推进创新综合体项目。苏南国家自主创新示范区加快“一区十四园”建设，充分发挥在镇高校作用，重点打造江苏科技大学海洋装备研究院等公共平台。支持丹阳、句容创建国家级经济开发区，扬中创建国家级高新区，句容、丹徒创建省级高新区。优化沿江、沿河、沿路、沿线产业布局，着力培育块状经济，提高产业集中度，推动20个产业“三集”示范园区提档升级，加快8个市级特色小镇建设，创成1个省级旅游风情小镇。

　　二是培育新业态。加快发展全域旅游。整合旅游资源，理顺管理体制，推进恒大童世界、魔幻海洋等重点项目，大力发展户外运动、水上运动、航空运动等新业态。繁荣文化创意产业。聚焦创意设计、数字出版、网络游戏、文化旅游四大板块，推进40个重点文化产业项目，举办第三届民俗文化周、第三届文化旅游创意产品设计大赛，力争文化产业增加值占GDP比重达5.5%。做强现代物流产业。实施“江海河联动、港产城融合”十大行动计划(22)，有效整合、高效利用港口岸线资源，培育港务集团等龙头企业。加快建设大港综合物流集聚区，打造镇江港总部物流基地。

　　三是运用新技术。实施创新型领军企业、高新技术企业、科技小巨人三大培育计划，新认定国家级高新技术企业100家，新增新型研发机构5家以上。推动规模以上工业企业专利全覆盖和高新技术企业发明专利全覆盖，万人发明专利拥有量达25件。加快丹阳（眼镜）知识产权快速维权中心建设。支持句容、扬中创建国家知识产权强县工程。

　　四是拓展新模式。扎实推进制造业智能化、绿色化、服务化、高端化，引进培育标杆企业。积极探索“互联网+商贸流通”，重点支持眼镜、汽车零部件、灯具等优势产业拓展电商交易平台，实现线上线下融合发展。加快发展电商楼宇经济，打造大禹山创意产业园、高创中心等一批优质载体。大力发展会展经济、平台经济、分享经济。

　　发展“四新”经济，一方面，必须高度重视人才支撑。人才是发展的第一资源。人才在哪里，希望就在哪里；顶尖人才在哪里，发展高地就在哪里。深入实施“金山英才”计划，加快领军人才创新驱动中心镇江分中心建设，举办首届创新创业人才博览会、第二届宁镇扬在地高校产学研合作周。新增国家“千人计划”15人，入选省“双创计划”50人，培育引进本科以上毕业生1.5万人。大力发展职业教育，积极开展职工培训，培养高技能人才1万人。另一方面，必须切实加强政策支持。认真落实省科技创新“40条”和市聚力创新“6条”，积极营造优良的创新生态。深化科技体制改革，扎实推进科技金融、区域创新等10个国家和省级试点项目，技术合同交易额力争突破1亿元。深化人事制度改革，完善人才培育、引进、使用的体制机制，通过提供人才公寓、薪酬补贴等方式，激发各类人才的创新、创业活力。

　　（三）着力深化供给侧结构性改革

　　这既是重大政治责任，也是重大发展机遇。供给侧结构性改革深化的过程，就是区域发展分化的过程。按照中央的部署要求，推动“三去一降一补”取得更大进展。

　　“去”得更坚决。淘汰落后过剩产能。实施40个去产能项目，去除钢铁行业产能70万吨，压降水泥行业过剩产能70万吨。对“地条钢”等非法生产经营行为保持高压态势。对扭亏无望的“僵尸企业”及时“止血”出清。促进房地产市场平稳健康发展。坚持去库存和稳市场并重，注重因地施策、分类施策。加强经营性用地调控，规范土地市场，合理把握土地供应节奏。全市商品住房去化周期保持在13个月以内，通过商改住、售改租多管齐下，将商办用房去化周期逐步压降至合理区间。防控化解区域性金融、债务风险。认真贯彻国务院《关于积极稳妥降低企业杠杆率的意见》，进一步扩大直接融资特别是股权融资比重。妥善处置企业债务和互联互保风险，强化互联网金融风险专项整治。加强政府性债务管理，完善国资监管体制，推动四大产业集团市场化转型，坚决守住不发生区域性系统性金融风险的底线。

　　“降”得更有效。全面落实国家和省、市降低企业成本的政策措施，进一步降低企业税费负担、制度性交易成本、用能成本、物流成本，为企业直接减负100亿元左右。

　　“补”得更精准。突出生态、生产、生活三个方面，认真实施省五大领域补短板专项工程，完善项目储备和推进机制，重点在增强公共服务功能、加强基础设施薄弱环节、强化民生保障和生态建设等领域加大投入、补好“短板”。

　　（四）着力推动农业提质增效

　　认真贯彻中央一号文件精神，深入推进农业供给侧结构性改革，促进农业增效、农民增收、农村增绿。

　　调整产业结构。大力发展优质粮油、高效园艺、特种养殖、碳汇林业、休闲农业五大特色产业，新增国家级高标准农田6万亩、高效设施园艺面积2.5万亩。加快30个重点农业园区建设，优化农业区域布局。深入实施科技兴农战略，强化农业新技术、新产品、新装备的创新应用，加快建设国家农业科技园区。健全农产品质量安全监管追溯体系。严格落实粮食安全责任制。完成第三次全国农业普查。

　　优化生产方式。全面推广戴庄模式，加强农村专业合作组织建设，打造戴庄经验示范村10个，新增市级以上示范家庭农场50家、农民专业合作示范社30家。积极发展创意休闲农业和乡村旅游，建成省级以上休闲农业示范点40个。支持“农联·亚夫在线”等农村电商发展，推动快递下乡。探索耕地轮作休耕制度试点，实施化肥农药零增长行动，大力开展山水林田湖整体保护、系统修复和综合治理，推动农业可持续发展。

　　夯实基层基础。把基础设施建设的重点放在农村。完善农田水利设施，加快新孟河延伸拓浚、九曲河整治。完成农村公路提档升级130公里。加快农村光纤宽带网络、4G通信网络、无线局域网络建设，推进农村互联网提速降费。深入实施村庄环境改善提升行动，建成1个美丽宜居镇、10个美丽宜居村庄。创新基层治理，高度重视农村“三留守”(23)问题，深入推进平安村庄建设，切实维护农村社会和谐稳定。

　　激发农村活力。深化农村土地“三权分置”改革，落实集体所有权，稳定农户承包权，放活土地经营权。土地确权登记颁证工作通过省级验收。依法推进农村土地经营权流转。稳步推进农村集体产权制度改革，建成农村产权交易中心，扎实开展30个村集体经济资产股份制改革试点。做好农村土地征收、集体经营性建设用地入市、宅基地制度改革和“两权”抵押(24)试点工作。

　　（五）着力深化改革开放

　　改革开放是决定发展的关键一招。更加积极主动抓改革、扩开放，为镇江的发展开辟新空间、注入新动力。

　　深化行政审批制度改革。深入推进“放管服”改革(25)，推动权力清单和互联网政务深度融合。建立“多规合一”“多评合一”联动机制，优化完善“e办事”平台，将服务内容扩大到全部审批事项和公共服务事项，范围延伸到所有乡镇，做到“应上尽上、全程在线、覆盖全市”。深化商事制度改革，推动“多证合一”。加快建立以信用为基础的监管执法体系，实现“双随机、一公开”(26)监管全覆盖。

　　统筹重点领域改革。深化生态文明综合改革，围绕中央“1+6”重点改革措施(27)、省22项主要任务，在环境准入、生态保护、市场机制、排污权、绿色金融等领域制定标准，并争取成为国家标准。深化投融资体制改革，筹建市属金融控股集团。深化教育领域综合改革，探索中小学校长职级制、职业教育混合所有制改革，积极推进教师“县管校聘”。深化价格机制改革，加大清费减负力度。

　　加快推动双向开放。落实国务院《关于扩大对外开放积极利用外资若干措施》，积极对接“一带一路”、长江经济带等国家战略，改革招商体制，完善考核机制，完成产业类实际利用外资13.5亿美元。实施外贸优进优出战略，重点培育中林、润华等10家进出口基地型企业。成立“走出去”服务中心，推动有条件的企业开展跨国并购。

　　（六）着力提升城市品质

　　城市发展是为了让生活更美好。城市大小不是主要目标，功能完备、舒适便利、环境优美才是关键。着眼于宜居宜业宜学宜游，深入推进“精美镇江”建设，让城市彰显镇江特色，讲好镇江故事，体现镇江品位。

　　规划更科学。尊重城市发展规律、尊重自然生态环境、尊重历史文化传承、尊重群众诉求，走“节约紧凑、精明增长、绿色发展”的路子。统筹各类空间规划，统筹“地、水、绿、文、美、产、居”多种要素功能，按照“生产空间集约高效、生活空间宜居适度、生态空间山清水秀”的总体要求，谋篇布局、统领全局，坚决防止“摊大饼”“铺摊子”，实现建一片、成一片、兴一片。

　　路网更通畅。扎实推进宁镇扬一体化，加快连镇铁路建设，开工建设宁句S6线，完成宁镇城际线路方案研究论证，开展镇扬过江通道前期可行性研究。加快市域一体化，启动镇丹、镇句快速通道建设，完成丁卯桥路快速化改造，打造市与三个辖市“半小时交通圈”。对原312国道、团山路西段实施城市化改造，构建市区环形路网。

　　功能更优化。随着路网骨架的拉开，着力往里面添人气、添内容、添功能。将中心城区划分为若干单元，针对文化、教育、体育、卫生、邻里中心等服务内容查漏补缺，统一规划布局，分步实施到位。加快推进棚户区城中村改造，老城区重点推进小规模、渐进式、微循环的有机更新和城市修补；新城区重点补齐公共服务等功能“短板”，吸引人气和商机。全面完成海绵城市建设三年试点任务，建成海绵主题公园、江苏大学海绵校园。大港水厂一期工程建成运行；完成云林、华山等输变电工程；改造老小区燃气立管1万户。

　　文脉更清晰。历史是城市之“根”、文化是城市之“魂”。深入研究镇江的历史文化，启动历史文化名城保护规划修编。加强城市设计，在传统历史文化中挖掘城市内涵和特有基因，打造一批独具镇江魅力的特色建筑。推进铁瓮城遗址、新河街、伯先路、大龙王巷历史文化街区建设，恢复一批名人故居，系统保护古镇、古村落和各类文化遗产，当好古城的守护者和历史文化的传承者。

　　管理更精细。积极运用大数据、云计算等技术管理城市。继续推进城市环境综合整治。实施新一轮环卫保洁市场化运作。按照“减量化、资源化、无害化”原则，扩大垃圾分类处置试点。深入开展占道经营、渣土扬尘、户外广告、违法建设四大专项整治，为广大市民创造更加干净、整洁的生活环境。

　　（七）着力保护生态环境

　　生态环境是最公平的公共产品、最普惠的民生福祉。以“263”专项行动为抓手，下更大决心推进生态建设。

　　打响“低碳”品牌。重点抓好“一区、一园、一岛、一镇、一院、一所、一平台、一中心、一基金”。加快建设生态城镇化示范区，编制启动区南片区规划，实施上湖湿地生态化改造。依托中瑞镇江生态产业园，积极招引低碳环保产业入驻。扎实推进扬中绿色能源岛建设，全面实施“金屋顶”计划(28)，全市新建分布式光伏发电装机容量120兆瓦。高标准打造低碳小镇。推进“生态云”平台升级。组建镇江低碳产业技术研究院和镇江国际低碳发展研究中心，建成低碳交通、低碳建筑等专业研究所。积极争取设立碳排放权交易所。着力放大低碳产业基金效应。精心举办第二届镇江国际低碳技术产品交易展示会。

　　做深“山水”文章。山水是写在大地上的文章，是大自然对我们的馈赠，我们必须善待。加快编制《山体保护导则》，严格控制开发建设。完成南山白龙山片区滑坡治理，推进黄鹤山、磨笄山等山体整治，抓好废弃宕口、轧石场生态修复。对主城区范围内已完成整治的山体，按照“一山一景”原则，实施美化亮化；对尚未整治的山体，开展退房还绿和生态修复，使其成为老百姓家门口的公园。按照“共抓大保护”要求，加强长江镇江段水质保护和生态建设，加快构建长江生态安全带。深入推进“一湖九河”水环境整治，完成西团结河、一夜河综合整治，打造古运河风光带。加强水源地保护，对化工园区之外的长江沿岸重点规划区域、京杭大运河沿岸1公里范围内的化工企业，启动关停搬迁工作。建成谏壁污水处理厂一期，完成镇江新区第二污水处理厂扩建。全市地表水国考断面优于Ⅲ类水质比例75%以上，太湖流域国考断面达标率达71.4%，集中式饮用水源地水质达标率保持100%。

　　彰显“花园”魅力。按照“彩色化、珍贵化、效益化”的要求，构建点、线、面相结合的绿化体系。加强高速出入口整治，打造整洁有序、美观靓丽的城市门户。完成焦山路沿线环境整治出新，构建滨江沿河风光带、主要国道、省道、高速公路和铁路沿线林带。丹徒区、京口区、润州区各新建一处街头绿地或垂直绿化一条街。鼓励社会参与，建设一批花园式街道、社区、学校、医院。市区新增绿地3000亩，建成区绿化覆盖率达43%。加强农村河塘、沟渠、道路绿化，逐步形成“道路林荫化、农田林网化、城市森林化”的城乡绿化格局。

　　狠抓“源头”防治。坚决打好治水、治气、治土三大攻坚战。深入推进“三个片区”综合整治，谏壁片区加快索普化工基地和新区化工园区周边居民搬迁，西南片区推进扬尘治理等23项重点任务，高资片区建立区域环境质量长效管理机制。完成重点化工园区、重点企业废气排放源整治，实现大型燃煤机组超低排放改造全覆盖。全年空气质量优良天数比例进一步提高，PM2.5浓度持续降低。积极开展土壤修复治理，建立重污染地块名录和开发利用负面清单。

　　（八）着力改善群众生活

　　只有让人民群众有更多的获得感、幸福感，我们所做的工作才会有认同感、归属感。

　　增加群众收入。实施就业优先战略，深化“创业镇江”行动，鼓励本地大学毕业生在镇创业就业，吸引外来“创客”落户镇江，扶持创业7000人、带动就业3.5万人。聚焦茅山老区，深入推进“百村万户”达新标、精准帮扶特困家庭奔小康、万个党支部结对帮扶万户特困家庭三大行动，确保经济薄弱村和低收入人口达标率60%以上。实施教育、健康等专项扶贫，着力解决好因病因残致贫返贫问题。完善社会保障和社会救助体系，大力发展社会福利和慈善事业，切实保障困难群体生活。

　　优化公共服务。完善新一轮教育布局规划，创建2个省级标准化社区教育中心，完成20个乡镇、学校教育现代化“双创”(29)工作，推动镇江高专“专升本”。整合市区医保经办机构，推进医疗、医保、医药“三医联动”；将世业镇打造成“全国健康镇”。扩大养老服务供给，促进养老服务产业发展。认真筹办中国户外运动大会、第二届江苏航空体育旅游季等体育赛事。广泛开展全民阅读，深入推进“书香镇江”建设，提高全民科学文化素质。

　　加强社会治理。创新社区治理方式，进一步完善“网格+网络”社区管理服务体系。深化“大爱镇江”建设，着力激发社会组织活力，更好发挥志愿者的作用。认真实施“七五”普法规划，建成市级公共法律服务中心。扎实推进八创全国双拥模范城。依法管理民族宗教事务。完善危化品防范处置“五大体系”，开展危化品、烟花爆竹、交通运输等重点行业专项治理，对发生较大以上安全生产责任事故的实行“一票否决”。完善社会治安防控体系，全面推进“阳光信访”和“法治信访”，营造和谐稳定的社会环境。

　　各位代表，保障和改善民生是政府工作的根本出发点和落脚点。今年我们将紧紧围绕全市人民所思、所想、所需、所盼，扎实办好10件民生实事：

　　1．推动精准扶贫。按照乡镇不低于100万元、有条件的村不低于50万元的标准，建立镇村扶贫基金。对城镇低保人员、特困职工、困难残疾人等实行社保补贴，对中断社保人员续保提供补贴。市、区两级财政安排的社会医疗救助补贴标准，分别由每人每年6元提高到每人每年15元。

　　2．完善教育设施。加快高校园区建设，推进市特教中心易地新建工程，新、改、扩建中小学校（幼儿园）33所，建设智慧校园30所。

　　3．提高医保水平。全面实施提高大额医疗费报销比例、完善补充医疗保险制度、拓展个人二级账户使用等八大医保便民惠民措施，稳步提高群众医保待遇。

　　4．实施文化惠民。积极推进文化场馆建设，建成20个生态文化广场，举办艺术知识普及公益讲座300场。

　　5．推进全民健身。打造市区“沿江、沿河、沿山、沿路”体育带，建成5个体育小公园，新建和提升70条健身路径，推动学校体育场馆向社会有序开放。

　　6．改善居住条件。推进山巷、和平路西等24个棚户区改造，实施2.6万平方米危房解危、1万平方米街巷改造；基本建成保障房2000套，货币化安置4000套。

　　7．方便市民出行。打通太古山路等断头路；新增200辆新能源公交车；新建国信南广场、市儿童医院等9个停车场，市区新增泊车位1300个。

　　8．加强养老服务。新建3个老年人日间照料中心，扩大城市社区老年助餐点服务范围；全市护理型养老床位占养老机构床位总数的50%以上。

　　9．提升社区服务。新建改造4个社区服务中心；在南徐新城片区开展“互联网+社区便利店”的倍全模式试点，解决群众“买菜难、卖菜难”。

　　10．打造“放心餐桌”。建成餐饮安全示范区，争创省级食品安全城市，中小学、托幼机构食堂良好以上等级90%以上。

四、加强政府自身建设

　　新一届政府肩负崇高的使命，承载人民的重托，必须牢固树立政治意识、大局意识、核心意识、看齐意识，严守政治纪律和政治规矩，坚决维护党中央权威，确保政令畅通、令行禁止；必须坚持“以人民为中心”的发展思想，忠实履行政府职责，为人民服务，对人民负责，让人民满意！

　　牢记为民宗旨。把执政为民贯穿政府工作全过程。加快转变政府职能，创新公共服务供给方式，完善政府购买服务清单，集中更多财力用于公共服务和民生改善。坚持放权于企、让利于民，正确处理好政府与市场的关系，让政府有形之手、市场无形之手、市民勤劳之手同向发力。大力弘扬“店小二”精神，深入农村、社区和企业听民意、接“地气”，办实事、解难题。

　　严格依法行政。坚持领导干部带头学法尊法用法，坚持运用法治思维和法治方式推动工作，坚持法定职责必须为、法无授权不可为，把政府活动全面纳入法治轨道。坚决贯彻市委决策部署，认真执行重大行政决策程序规定，确保决策制度科学、程序适当、过程公开、责任明确。依法接受人大及其常委会的监督，自觉接受人民政协的民主监督，认真做好人大议案、代表建议和政协提案办理工作。主动接受司法机关监督，接受社会公众监督和新闻舆论监督，充分听取民主党派、工商联、无党派人士和各人民团体的意见。深化政务公开，完善新闻发布制度，让权力在阳光下运行。

　　切实改进作风。建立高效运作机制，政府领导班子坚持集体领导、分工负责、相互协作。坚持求真务实、真抓实干，不搞虚假政绩、不搞数字攀比、不搞形象工程。少讲正确的废话，多讲有用的真话；少开无效的会议，多办有效的实事；少出空洞的文件，多出管用的办法。“军中无戏言”，承诺必兑现。对市委定下的事、向群众承诺的事，说则必干、干则必成。大力弘扬担当精神，积极营造想干事、能干事、干成事的氛围，不断提振公务员队伍的精气神。严格机关绩效管理，健全问责处罚机制，力戒敷衍了事、推诿扯皮、为官不为，坚决整治庸政、懒政、怠政。支持和帮助各辖市区、镇江新区、镇江高新区解决事关长远发展的重大问题。尊重基层实践，尊重群众首创精神。

　　坚持廉洁从政。严格执行党内政治生活若干准则、党内监督条例和问责条例，严格执行党风廉政建设责任制，切实履行主体责任。认真落实“一岗双责”，深化廉政风险防控，坚持不懈纠正“四风”。坚持用制度管权、管事、管人，强化权力运行制约监督，加快形成不敢腐、不能腐、不想腐的体制机制。强化行政监察和审计监督，严管公共资金、严管公共资源交易、严管国有资产资本。坚持无禁区、全覆盖、零容忍，严查重点领域和关键岗位腐败，严惩侵害群众利益的不正之风和腐败问题。

　　各位代表，人民给我们鼓劲，我们更要使劲。我们将在市委的坚强领导下，以一往无前的钻劲，适应新常态，拓展新空间；以一着不让的拼劲，抢抓新机遇，推动新发展；以一抓到底的韧劲，破解新难题，实现新突破。用实干、实效、实绩，回报全市人民的信任和重托，迎接党的十九大胜利召开！

　　上下同欲者胜。镇江的明天一定会更美好！
